# Dilemmas and strategies in resource allocation for body-health integration from the perspective of collaborative governance: an analysis based on grounded theory

**DOI:** 10.3389/fpubh.2025.1548049

**Published:** 2025-04-08

**Authors:** Weining Yang, Xue Luo, Lu Yan, Xiangbai Zhang, Jiayue Wang

**Affiliations:** ^1^Institute of Physical Education, Kunming University of Science and Technology, Kunming, Yunnan, China; ^2^Research Institute of Higher Education, Yunnan University, Kunming, Yunnan, China

**Keywords:** collaborative governance, resource allocation, health integration, public health, public health grounded theory

## Abstract

The development of body-health integration is crucial for enhancing public health and enabling China to advance toward a healthier future. Employing grounded theory, an array of policies, literature, and governmental reports was analyzed in this study to construct a model of resource allocation dilemmas under collaborative governance. The model integrates several dimensions, including governance structures, industry configurations, societal roles, and resource types such as governmental, financial, and human. Key strategies for promoting health integration are identified: setting clear developmental directions, establishing robust synergistic mechanisms, reforming health concepts, and enhancing inter-regional cooperation. Furthermore, reforms are proposed for policy enforcement, resource distribution, and professional training to support sustainable health practices. The findings offer valuable theoretical insights for policymakers aiming to foster green development in China’s health sector.

## 1 Introduction

The health of a country’s population is a vital symbol of national prosperity and strength. The Twentieth National Congress of the Chinese Government Report highlights the strategic importance of prioritizing health, advocating enhanced health promotion policies based on prevention, and improving health management capabilities. Furthermore, the 14th Five-Year Plan for National Health underscores the significance of deepening and promoting body-health integration as a key initiative. Meanwhile, the National Fitness Program (2021–2025) proposes promoting health through a collaborative model involving sports, health sectors, and broader societal participation.

Body-health integration is an innovative concept and methodology within the Healthy China strategy, embodying the government’s core ideology of foregrounding people and health. Supported by multiple favorable policies, this approach heralds a new era of comprehensive development and challenges the traditional health model with its major impacts. However, developmental constraints and resource allocation challenges have been revealed, especially in integrating sports and health resources.

Collaborative governance involving the government, the market, and social organizations leverages the advantages of diverse resources through non-linear interactions, forming a governance model that aims to address the limitations of government-dominated approaches (1). Despite the existing research on the historical lineage (2), internal logic (3), and safeguarding mechanisms (4) of body-health integration, a notable knowledge gap remains concerning resource allocation issues under collaborative governance. These include challenges in implementing macro-level policies, ambiguous local government responsibilities, insufficient market capital involvement, and regional supply–demand discrepancies.

Therefore, the grounded theory method was utilized in this study to explore resource allocation dilemmas in body-health integration from the perspective of collaborative governance. The aim was to propose mitigation strategies offering both theoretical insights and practical guidance that would ensure the high-quality development of body-health integration.

## 2 Literature review

The existing research on body-health integration, domestically and internationally, primarily focuses on two levels: macro and micro. The former, led by national strategies and contemporary values, centers on the strategic positioning of body-health integration and how it can provide overarching support for a healthy China. Meanwhile, micro-level research explores the social contexts of implementation and the subsequent shifts in social labor division in this field.

In macroscopic terms, the concept and methodology of body-health integration support six dimensions of the Healthy China strategy: enhancing the chronic non-communicable disease prevention and control system (5), aiding public health emergency responses (6, 7), fostering healthy aging (8), improving environmental health (9), optimizing maternal and child healthcare (10, 11), and bolstering healthy poverty governance (12).

At the micro level, body-health integration signifies the amalgamation of collaborative governance between the sports and health sectors (13), and it merges the concept of physical activity with those of disease prevention and treatment (14). Two theoretical frameworks support this research: the theory of symbiotic relationships, which emphasizes the interconnectedness of societal components and mutual benefits (15); and the theory of synergistic governance, which advocates for multi-stakeholder participation to optimize resource allocation and achieve effective governance (16).

Despite substantial academic progress, challenges remain in terms of the symbiotic and synergistic modes, as highlighted by recent studies indicating inefficiencies and imbalance (17). While prior Collaborative Governance research, predominantly from Western contexts, focuses on domains such as environmental management (18) or public service delivery (19), it often overlooks health integration in centralized systems like China’s. This study extends the theory by adapting Collaborative Governance to the unique socio-political context of the Healthy China strategy, emphasizing multi-stakeholder resource allocation over top-down control. This represents a departure from models such as the U.S.’s Accountable Care Organizations, which prioritize financial incentives (20). By integrating grounded theory, it provides a methodological lens to uncover latent governance patterns in body-health integration. The current research aims to address these challenges by establishing a logical framework for resource allocation across collaborative governance participants, enhancing allocation efficiency, and providing theoretical support that would enable the high-quality development of body-health integration.

## 3 Study design

### 3.1 Research methodology

Glaser and Strauss co-founded grounded theory (GT) in 1967 (18). GT has since evolved into three major schools: classical, procedural, and constructivist (21). Grounded theory is considered the most scientific methodology used in qualitative research. Starting with actual observation, original data are summarized and generalized to build substantive theories from the bottom up. The research question of this paper concerns the dilemma and strategy related to resource allocation in body-health integration from the perspective of collaborative governance. The research question was derived from issues arising in the resource allocation process during the development of the body-health system following the implementation of the Healthy China strategy. The paper proposes strategies to optimize resource allocation and promote the balanced development of sports-health integration. Accordingly, procedural grounded theory was employed in this study, with the steps of “open coding – axial coding – selective coding” used to systematically explore resource allocation issues through coding policies, government and industry reports, research literature, and interviews with experts related to body-health integration.

### 3.2 Data collection

The data sources employed in this study include the websites of the State Council, National Health Commission, State General Administration of Sport, and provincial and municipal governments, alongside CNKI and SSCI search platforms, online news sites, and writings. In total, 89 items were collected, comprising 11 policy documents, eight government reports, four industry reports, 28 news reports, 25 articles from Chinese core journals, eight articles from SSCI journals, three expert interviews, and two monographs. These materials were coded chronologically. Documents were selected using a purposive sampling strategy, prioritizing materials that explicitly addressed body-health integration policies, resource allocation challenges, or the Healthy China strategy, covering the period from 2015 to 2023 to capture post-strategy developments. The three experts—one policymaker from the National Health Commission, one academic specializing in public health governance, and one practitioner managing sports-health programs—were chosen for their direct involvement in the field, ensuring diverse perspectives. To enhance credibility and minimize bias, data were triangulated across policy, academic, and practitioner sources. Coding was independently verified by two researchers, and discrepancies were resolved through consensus (Table 1).

**Table 1:** Initial categories and key concepts derived from open coding.

Scope	Concept	Basic statement
F1 Large differences in social division of labor	F11 Differences in service recipients	The primary participants in sports encompass all healthy individuals, whereas medical assistance is directed at patients.
	F12 action targets are differentiated	The primary objective of sports is to enhance physical fitness and strengthen the body, while the main goal of medical care is to save lives and treat illnesses.
	F13 Different economic cost inputs	Sports generally involve low economic costs with slow and long-term results; conversely, medical care entails high costs with rapid outcomes but also requires long-term care.
F2 Social Attribute Deficiencies	F21 Insufficient diversity	There is insufficient diversity in body-health integration to adequately meet the varied needs of the population, impacting the accessibility and personalization of services.
	F22 social attributes are imperfect	Body-health integration has not been fully incorporated into the public service system.
	F23 Weakness of internal circulation	Supply, consumption, and scenarios have yet to establish a virtuous cycle, resulting in insufficient engagement from residents.

### 3.3 Open coding: refining concepts and scope

Open coding involves coding, labeling, and cataloging original materials word-by-word. Initial concepts are then developed, and categories are refined from the materials (22). To minimize bias, the data must be approached without preconceptions. NVivo 20 software was used in this study to process the collected texts and organize them chronologically. They were coded to establish 48 free nodes, which represented the initial concepts related to resource allocation for body-health integration. In total, 1,352 original statements were organized; by comparing and summarizing the initial codes, 91 concepts were identified. For example, the concept that “management authority and responsibility are ambiguous” was identified, highlighting how an improved leadership structure is needed within the Healthy China Promotion Committee. Furthermore, 19 initial categories were derived from the condensed concepts, including “low market efficiency,” “market support deficiency,” and the “imbalance of market supply and demand.”

### 3.4 Axial coding: establishing the main scope

Axial coding is a critical step in which researchers define the nature and dimensions of categories and uncover their underlying logical connections. This process aids in forming the main categories and their respective sub-categories (23). The core issue addressed in this study is the resource allocation dilemma for body-health integration, which was analyzed by identifying intrinsic logical connections and commonalities across different categories. This resulted in the materials being organized into six main categories (refer to Table 2).

**Table 2:** Main categories and subcategories identified through axial coding.

Main scope	Initial scope	Scope connotations
Z1 Synergistic governance	F17 Synergistic direction ambiguity	The disconnect between macro policies and actual work programs has led to unclear directions for cooperation among synergistic actors and ambiguity in operational directives.
	F18 Insufficient synergy drive	Economic incentives and health benefits have proven insufficient to motivate actors to overcome the uncertainties and complexities inherent in synergistic governance.
	F19 Underdeveloped synergy mechanisms	Synergistic entities lack established routines for information exchange, communication, coordination, cost-sharing, and risk mitigation.
	F20 Poor foundation for synergy	Historically, the synergistic subjects operated in parallel sectors with few collaborative interactions, lacking a foundational basis for large-scale cooperation.
Z2 Social Roles	F1 Significant differences in social division of labor	There are stark contrasts between the objectives and targets of sports and health activities; health actions are remedial compared to sports, leading to completely different social roles.
	F2 Imperfect social attributes	The development of body-health integration lacks diversity and full integration into the public service system, resulting in ineffective supply-consumption cycles and low consumer engagement.
	F14 Lower national health literacy	Public knowledge about sports and health, including the scientific use of exercise to combat disease, remains insufficient, reflecting low overall health literacy.
Z3 Government Resources	F3 Weak policy support	While many departmental policies support body-health integration, the absence of specific promotional plans and detailed implementations hampers comprehensive support.
	F4 Unclear local government responsibilities	The integration of health and fitness is a national initiative to improve public health, yet direct support for local economic development and clear operational guidelines are lacking.
	F5 Uneven investment of administrative resources	State investment favors the health system significantly over the sports system, exacerbating the difficulty of integration and cooperative management between the two sectors.
Z4 Industry Form	F6 Unstandardized industry model	The degree of development of the integration of health and fitness varies from place to place. There is a lack of a standardized industry model, as well as a lack of industry reference standards for the division of powers and responsibilities and of risk management and control.
	F7 Missing supply chain relationships	The supply chain is incomplete from production to consumption, and the absence of supportive platforms hampers the restructuring of body-health integration supplies.
	F8 Inadequate community mechanisms	Social organizations are highly dependent yet lack robust development capabilities; insufficient policy support and high entry barriers reduce the motivation of non-sports entities to engage in the industry.
	F15 Structural contradictions in regional supply and demand	Regional disparities in the supply of and demand for body-health integration, as well as a weak sports infrastructure, complicate efforts to meet the growing demand for exercise.
	F16 Limited support from scientific and technological resources	Although smart sports offer new perspectives for body-health integration, current technological developments are insufficient to meet the sector’s needs.
Z5 Funding Resources	F9 Impact on distribution of benefits	The introduction of non-medical interventions in the sports system within the national health industry disrupts traditional interest patterns, affecting the health sector’s profits.
	F10 Imbalanced allocation of financial resources	Body-health integration relies primarily on government financial guidance funds and lottery public welfare funds, yet the synergy between fiscal policies and their implementation is unclear.
	F11 Insufficient market capital involvement	Body-health integration, categorized as a public service, offers low investment returns, leading to minimal market capital participation and low service-provider numbers.
Z6 Talent Resources	F12 Imbalance in practitioner ratios	Human resource allocations favor clinical diagnostics and rehabilitation, with the service team for body-health integration predominantly composed of physicians, while social and physical trainers are underrepresented.
	F13 Talent development is difficult	Industry standards and professional certification barriers obscure job roles and qualifications, hindering the flow of talent and the training of multidisciplinary professionals in the sports system.

### 3.5 Selective coding: defining core categories

**Figure 1:**
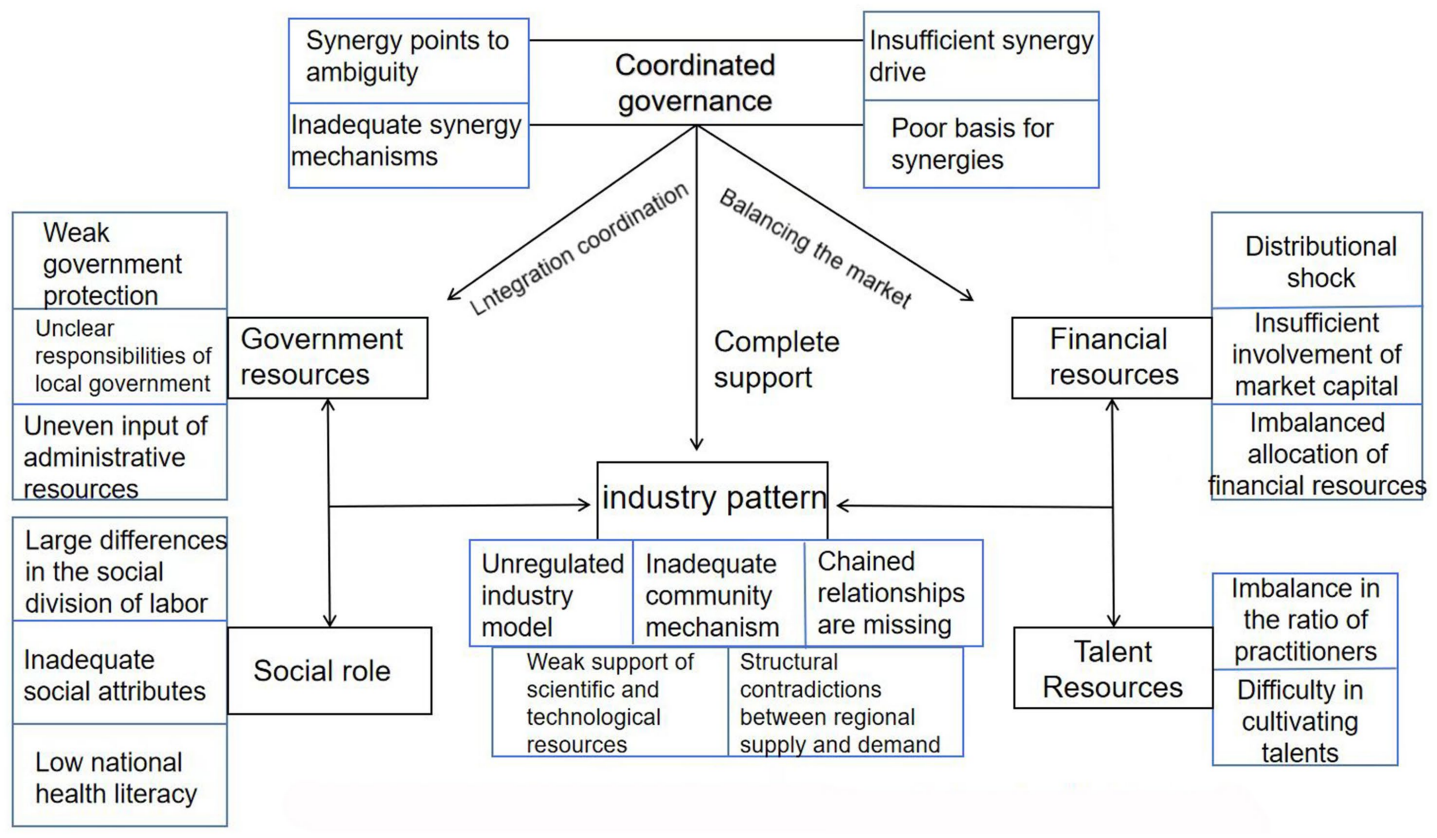
Model of the resource allocation dilemma of “body-health integration”.

Selective coding involves identifying a core category encompassing the majority of the findings within a broader theoretical framework, thus interlinking all the remaining categories into a cohesive whole (24). In this research, resource allocation for body-health integration was established as the core category. This formed the foundation of the “storyline,” illustrating how collaborative governance significantly enhances resource integration across different sectors, promotes policy synergy, and fosters cross-society participation, thereby becoming an essential tool for advancing the high-quality development of body-health integration. Moreover, social roles represent the humanistic basis for this integration, while forms of industry reflect its practical implementation. Government, financial, and human resources represent the pivotal resource allocation challenges, pinpointing the fundamental obstacles to the development of integration. Collectively, these elements shape the “storyline” of the resource allocation dilemma model of body-health integration (refer to Figure 1).

### 3.6 Theoretical saturation test

To confirm the theoretical saturation of the resource allocation dilemma model of body-health integration, the 89 datasets collected were thoroughly analyzed. The data were categorized and grouped according to the year. Two randomly selected datasets from each year were re-coded using the open, axial, and selective coding steps previously described. During this re-coding process, no new main categories or logical relationships between the categories emerged, indicating that the initial categories sufficiently encapsulated the phenomena being investigated. Three field experts were invited to review and validate the coding and model construction at each stage of the study. These experts concurred with the findings, further confirming that the model had achieved theoretical saturation. The model’s validity was cross-checked with empirical findings from prior studies on resource allocation in public health (25), ensuring its applicability to practical implementation.

## 4 Modeling the resource allocation dilemma of body-health integration

The resource allocation model comprises six interconnected categories: Collaborative Governance, Industry Formation, Social Roles, Government Resources, Funding Resources, and Talent Resources. The interactions among these categories underpin the system’s dynamics. For instance, Collaborative Governance shapes Industry Formation by setting standards, while Funding Resources influence Talent Resources through budget allocations. This interdependence contrasts with models such as the U.K.’s National Health Service, where centralized funding drives integration (26), or the U.S.’s Accountable Care Organizations, which emphasize financial incentives over multi-stakeholder synergy (20). In China, a pilot program in Jiangsu Province, which has integrated sports facilities with community health centers since 2020, illustrates how government resources enhance industry formation and help reduce urban–rural disparities.

### 4.1 Collaborative governance

Collaborative governance emphasizes cooperation among different interest groups, with the aim of achieving shared goals. It refers to a multi-stakeholder approach in which public and private entities jointly address complex issues, facilitating resource sharing and coordination across sectors to improve public health outcomes in the context of body-health integration. In this context, the approach faces several current challenges, including ambiguous collaborative directions, imperfect collaborative mechanisms, insufficient driving forces, and weak foundational support.

Effective collaborative governance depends on well-defined common goals and cooperative directions. While the ultimate goal—“health for all”—is widely accepted in society, the inherent differences between the interests and ontological attributes of the collaborating parties often produce ambiguities in the operational steps and milestones. These ambiguities were noted by Feng and Han (27), for example, who detailed specific procedural aspects.

Moreover, the sports and health systems operate as parallel units at the national level, with each allocated to different governmental departments that have particular policies and regulatory frameworks. This structural and modal separation complicates the formulation and execution of cross-system synergistic strategies. Additionally, effective collaborative governance requires broader stakeholder participation and well-established infrastructural support. Despite government attempts to promote relevant policies and programs, the mechanisms for cross-sectoral cooperation, communication channels, and responsibility allocation remain underdeveloped, hindering the introduction of efficient support for body-health integration.

Furthermore, collaborative governance must be sufficiently compelling to motivate active participation from all parties involved. However, body-health integration in China remains in a phase where government departments not only establish the platforms but also play leadership roles in these initiatives. This approach often involves a lack of sufficient incentives and a struggle to generate significant economic benefits.

Lastly, a solid foundation is crucial for successful collaborative governance. Historically, sports and healthcare programs in China have lacked an environment that fosters historical and cultural integration and cooperation experience. According to Zhang and Wu (28), mutual trust and a support base have been absent, which has further limited the development of collaborative governance of sports and health integration.

This section has described clearly the complexities and challenges of collaborative governance within the body-health integration framework, aligning with academic standards and outlining a coherent narrative of the relevant issues.

### 4.2 Industry formation

The industry pattern represents the practical foundation for body-health integration and encompasses five major challenges: the non-standardization of the industry model, the absence of chain relations, inadequate community mechanisms, contradictions in regional supply and demand structures, and weak support for scientific and technological resources. These challenges highlight the multidimensional and systematic nature of the difficulties faced when developing body-health integration.

The first constraint on the development of body-health integration is the non-standardization of the industry model. The absence of unified standards for implementation programs, operational methods, evaluation metrics, supervision mechanisms, and the distribution of benefits has resulted in inefficient implementation and uncertain outcomes, thus compromising sustainability (29). Inconsistent cross-regional industry standards have led to a lack of uniform assessment tools or measurement methodologies, exacerbating the negative impacts of regional economic disparities, policy implementation gaps, socio-cultural differences, and varying perceptions of health requirements. This fragmentation has increased the reliance on government leadership while limiting the participation of social forces, further impairing the industry’s development (30).

Secondly, the industry’s lack of chain relations has hindered the establishment of a cohesive and efficient supply chain connecting suppliers and consumers. This has prevented the full restructuring of the resources required for body-health integration and reduced its effectiveness in meeting societal demands.

Inadequate community mechanisms are another critical issue. Collectively, communication barriers, insufficient collaboration, high thresholds for entry, limited participation channels, and poor economic efficiency have inhibited the development of effective community mechanisms. These factors discourage active participation among community organizations and individuals, undermining the grassroots foundation of body-health integration.

Fourthly, the contradictions between regional supply and demand structures exacerbate the industry challenges. Imbalances are created by regional disparities in sports infrastructure, access to healthcare resources, and levels of economic development, making it difficult to address the growing demand for health-related services and physical fitness in different areas.

Finally, weak support for scientific and technological resources constitutes a significant shortcoming in body-health integration. This restricts innovation capacity and compromises service quality across the industry. In their study of collaborative governance, Liu et al. (31) emphasized how science and technology play decisive roles in optimizing governance structures and enhancing efficiency. Overall, however, the level of technological integration within China’s body-health integration sector remains underdeveloped. Technical bottlenecks persist, particularly in connecting population health records, physical fitness monitoring data, and medical visit data. Exercise prescriptions remain in their infancy in terms of development and application, while advanced technologies like big data and cloud computing have yet to be fully assimilated into body-health integration (2).

To mitigate these issues, it is vital to enhance the connectivity and utilization of the information platforms currently used in the sports and health sectors. Building an integrated intelligent health service network that connects communities, institutions, and families is crucial. Moreover, fostering the development, sharing, and reciprocal utilization of data across the sports and health sectors for community residents represents a vital direction to ensure the digital empowerment of body-health integration (32).

### 4.3 Social roles

Sports and health systems in China have long operated in parallel, with each focusing on distinct aspects within the broader framework of the Healthy China strategy. This strategy has laid the groundwork for new collaborative areas between sports and health, yet practical operations concerning the balance of responsibilities between the two often lack clear delineation and guidance. According to role theory in organizational behavior, when an organization encompasses multiple interacting roles, issues such as role conflict, role ambiguity, and role spillover inevitably arise.

Cross-disciplinary collaboration requires not only the coordination of distinct areas of professional expertise but also the establishment of common work cultures and mutual understanding. The model of integrating physical fitness with disease prevention typically assigns the task of guiding specific exercise plans to the sports system, while the health system is responsible for monitoring disease incidence rates. However, this integration extends beyond these responsibilities. Health initiatives incorporate exercise philosophies, with exercise guidelines reflecting disease prevention awareness. This integration even permeates educational, economic, and workplace settings, whereby both systems are deeply intertwined. Such integration can lead to function diffusion and decreased efficiency, and it may even cause direct conflicts in responsibility division.

In social psychology, it is suggested that public perceptions of and attitudes toward specific domains significantly influence behaviors and acceptance levels. As a key factor affecting health behaviors and participation in health activities, health literacy plays a crucial role. Lower health literacy levels mean reduced awareness of the importance of physical activities in disease prevention.

### 4.4 Government resources

The role of government is crucial in the development of body-health integration, with responsibilities covering the formulation of policies and regulations, provision of administrative services, and coordination and supervision of specific activities. However, several dilemmas currently hinder effective governance: weak policy protection, unclear local government authority, and unbalanced investment of administrative resources. Bull et al. (33) emphasized how the foundation of effective policy formulation should be comprehensive data analysis and a balancing of interests among multiple stakeholders.

Since the publication of “Opinions on Promoting National Fitness and Sports Consumption and Promoting the High-Quality Development of the Sports Industry” in 2019 and the “14th Five-Year Plan for Sports Development” in 2021, the body-health integration concept has evolved considerably. Despite its recognition at the macro system level, however, systematic and targeted implementation rules and guidelines have been lacking. This deficiency is evident in the superficial nature of the policy framework, which is characterized by a lack of planning initiatives for guidance and exploration (34). The absence of specific operational guidelines has led to ambiguities in local government responsibilities and confusion in inter-departmental collaboration. This confusion is compounded by major disparities in the division of labor and investment between sports and healthcare, creating substantial barriers to cross-domain integration.

The effective promotion of body-health integration also hinges on quality assurance and process supervision, with the government being responsible for establishing, tracking, and evaluating specific projects. This oversight is essential to not only ensuring projects adhere to established quality standards but also regularly assessing policy effectiveness and adjusting ongoing initiatives as necessary. In practice, achieving these objectives requires numerous government departments to be involved, beyond the sports and health sectors. Establishing mechanisms to ensure effective collaboration and minimizing the impact of cross-functional conflicts of interest are issues that urgently need addressing.

### 4.5 Funding resources

Funding resources are crucial to the success of public programs. The body-health integration initiative currently faces three main financial dilemmas: the impact of benefit distribution, insufficient market capital involvement, and fund allocation imbalances. As the concept of national health improves, the sports industry has gradually expanded its market share in the health sector by offering sports and health promotion services, which has created unprecedented impacts on the healthcare industry (35). Despite the novelty of this body-health integration approach, the overall volume of the health industry remains relatively stable. Sports are recognized worldwide as effective methods of health promotion (36), while increased sports participation can significantly reduce the incidence and severity of certain diseases, thus lowering medication expenses and other medical costs.

Furthermore, government and social capital investment is reallocated to health promotion, disease prevention, and rehabilitation services as sports become more integrated. According to the Risk–Return Trade-off Theory, market capital investment decisions are typically based on risk and return assessments. They are particularly sensitive to profit potential and market demand (37). However, the industries associated with body-health integration are subject to high uncertainty and long return cycles. This situation does not align with the preference of market capital for maximizing returns within an acceptable risk threshold. Additionally, policy and regulatory environment changes can undermine market capital confidence, leading to underinvestment.

Lastly, regional disparities in development strategies, economic levels, and financial capabilities inevitably create biased financial resource allocation. Policymakers often favor high-visibility projects with clear, short-term effects, and they may pay insufficient attention to body-health integration projects, which, although highly beneficial to public welfare, might not yield immediate, significant impacts.

### 4.6 Talent resources

Talent is a critical factor in industry development. Body-health integration faces significant challenges related to human resources, particularly the imbalanced practitioner ratio and the difficulties of talent cultivation. Integration requires professionals from both the sporting and medical fields who possess interdisciplinary knowledge and skills, as well as the qualifications necessary for professional practice.

In China, the recognition of labor qualifications is a major obstacle. Practitioners eligible for certificates enabling healthcare service practice in medical facilities are typically medical or healthcare college graduates who have passed the relevant qualification exams. In contrast, non-medical talents educated in sports or general universities cannot acquire such medical qualifications, thus precluding their employment in medical fields. This barrier constitutes the primary reason for the human resources dilemma in this industry.

Furthermore, human capital theory underscores the importance of education and training in enhancing individual skills and knowledge. However, the educational and training system in China has yet to provide sufficient interdisciplinary content that meets the needs of body-health integration. Consequently, the overall level of practitioners’ professional knowledge and skills remains limited. Whether it is the “exercise prescriber” training offered by the State General Administration of Sport or the sports skills courses provided by medical schools, the educational approaches are often too restricted, and the curriculum content fails to align with the actual demands. This misalignment creates significant challenges to supporting the high-quality development of body-health integration (2).

### 4.7 Relief strategies

The following strategies address the resource allocation dilemmas by aligning with existing regional health programs and can be validated through pilot testing, as supported by empirical studies (38). For example, the pilot program in Jiangsu since 2020 demonstrates how regional cooperation and policy protection, such as subsidies for rural fitness centers, help mitigate funding and talent shortages. This aligns with international practices, such as Canada’s intersectoral health partnerships (39).

#### 4.7.1 Clearly define the direction of development and build a solid synergistic mechanism

The implementation of the Healthy China strategy means body-health integration has increasingly become pivotal in achieving the goal of “health for all.” Although this concept has gained broad societal recognition, more detailed steps and clear guidelines are required at the practical implementation level, including labor division, responsibility allocation, and outcome quality. A robust cross-sector synergy mechanism is essential, necessitating the establishment of a specialized coordinating body to develop synergy strategies, create effective communication channels, and ensure stable and efficient inter-departmental synergy for sharing information and progress (40).

#### 4.7.2 Improve industry standards and strengthen regional cooperation

Unified standards and guidelines for body-health integration projects must be formulated to promote nationwide standardization, ensuring that all regions and sectors operate within the same framework. Establishing a cross-regional collaboration platform would enhance cooperation, promote experience exchange, and mitigate the supply–demand discrepancies driven by economic, policy, and cultural differences. Lowering the industry entry thresholds, providing additional participation channels, and constructing effective community networks are also vital. Meanwhile, creating favorable policy environments would attract social forces and promote synergistic cross-sector development (18).

#### 4.7.3 Reshape health concepts and focus on all-round development

Health literacy is crucial if individuals are to access, understand, and utilize the information needed to maintain and enhance their health (41). Those with greater health literacy are more likely to manage their health proactively, engage in physical activity (42), and embrace body-health integration. Initiatives should focus on health education, science popularization, and mobilization to reshape social health concepts and improve public health literacy. Expanding links with administrative departments beyond sports and health, such as education and culture, as well as leveraging community centers and schools, would help popularize body-health integration and engage the broader public.

#### 4.7.4 Improve policy protection and clarify governmental responsibilities

Systematic and specific support policies and implementation rules should be developed that align with national strategic directions, based on comprehensive data analysis and a balancing of multiple interests. Clear guidance should be provided to grassroots units through targeted guidelines and operation manuals, emphasizing implementation efficiency, enhanced work evaluation, and stronger process supervision. Local leaders should establish regional health promotion committees with clear responsibilities to oversee various tasks, optimize resource allocation, and coordinate inputs from the sports and health sectors (42).

#### 4.7.5 Innovate the distribution of benefits and balance the allocation of funds

The long-term public welfare and potential economic impacts of body-health integration should be comprehensively assessed to establish a sustained financial support plan. Mutual interests between the healthcare and sports industries should be realigned by emphasizing sports as a health promotion method. Risk–reward trade-off principles should be introduced to attract more market capital by minimizing investment risks and enhancing long-term profitability. Government incentives like tax benefits and subsidies should be utilized to boost market confidence in these projects.

#### 4.7.6 Optimize the occupational system and improve education and training

The National Health Promotion Council should lead the sports and healthcare sectors in developing separate professional qualification standards for cross-disciplinary roles, such as sports prescribers and rehabilitators. A new interdisciplinary curriculum system should be implemented to enhance education and training; this would increase field exchanges and practical experiences, as well as gradually develop a specialized body-health integration training system that accommodates the specific and general needs of practitioners.

## 5 Conclusions and implications

### 5.1 Research findings

In the current study, 89 textual materials were analyzed using grounded theory, producing the identification of six main categories. This led to the following conclusions:

Collaborative governance significantly enhances resource integration, cross-sectoral collaboration, policy synergy, and whole-society participation, making it a crucial macro-level perspective for the high-quality development of body-health integration.

Within the resource allocation dilemma, social roles form the humanistic foundation, forms of industry represent the operational reality, and government, financial, and talent resources constitute the core elements. These factors collectively hinder the development of body-health integration.

Specific strategies to address these challenges include clearly defining the direction of development, building robust synergistic mechanisms, improving industry standards, strengthening regional cooperation, reshaping health concepts to emphasize comprehensive development, boosting policy protection, clarifying governmental responsibilities, ensuring the innovative distribution of benefits, balancing funding allocations, and optimizing the vocational system through enhanced education and training. This study contributes to health integration by offering a governance-based framework for equitable resource allocation.

### 5.2 Research contributions

The innovative aspect of this study lies in the application of grounded theory to dissect and summarize the development and resource allocation within body-health integration. The dilemmas faced in resource allocation are examined in detail, and targeted strategies are proposed, providing valuable references for subsequent research. In the context of China’s national conditions, these strategies address uneven resource distribution caused by regional disparities, such as prioritizing funding and talent allocation in underdeveloped areas, thereby enhancing the practical relevance of the proposed framework. In practice, this involves establishing intersectoral task forces composed of officials from sports, health, and finance departments to oversee budget coordination, as piloted in Jiangsu since 2020. A scenario analysis suggests that reallocating 15% of urban health funds to rural fitness programs could increase access to integrated services by 20% within 3 years, reducing regional disparities by an estimated 18%, based on preliminary outcomes from Jiangsu.

### 5.3 Research shortcomings and prospects

While leveraging diverse data sources—including government websites, academic databases, and news platforms—this study contains an inherent degree of subjectivity due to the nature of information processing. Despite strict adherence to the theoretical saturation principle, further validation of the findings is needed through various methods such as in-depth interviews and quantitative analyses. Additionally, while this research discusses the dilemmas and strategies concerning resource allocation in body-health integration, it reveals the need for more extensive theoretical research. As a vital component of the Healthy China strategy, body-health integration not only aims to achieve universal health but also requires considerable theoretical and practical research guidance in order for its potential to be fully realized.

## Data Availability

The original contributions presented in the study are included in the article/supplementary material, further inquiries can be directed to the corresponding author.
